# Retraction: Sheltering of deleterious mutations explains the stepwise extension of recombination suppression on sex chromosomes and other supergenes

**DOI:** 10.1371/journal.pbio.3003106

**Published:** 2025-03-27

**Authors:** The PLOS Biology Editors

Following publication of this article [1], concerns were raised regarding the validity of the main conclusions and the support for the inferences about the sheltering process described within the article. The *PLOS Biology* article [1] builds on a prior finding that an inversion that suppresses recombination may spread if it is less deleterious than the average [2], and claims to demonstrate higher rates of spread to fixation of inversions on Y chromosomes compared to autosomes over a wide range of realistic parameter values owing to a Y chromosome never experiencing homozygosity (recessive deleterious mutations thus being “sheltered”). The sheltering model was thus claimed to potentially explain recombination suppression over broad parameter space. Concerns were raised about whether the claimed breadth of parameter values had been demonstrated.

Specifically, it was raised that the claim of a broad parameter space relies on Fig 3C, which employed a methodological approach that excludes inversions lost within 20 generations (as described within the figure legend of Fig 3C) and that analysis of a full origin to fixation model (including the first 20 generations) was not clearly consistent with the breadth claims [3]. In particular, when considering a full origin to fixation model for neutral mutations (s =  0), a case in which sheltering could not occur, results for much parameter space are similar to those claimed to support the sheltering process [3], thus questioning whether the inference of breadth of parameter space where sheltering was the causal process (as opposed to drift) was valid.

*PLOS Biology* discussed these issues with the authors, who stated that when analyses include the first 20 generations, the absolute frequencies of fixation are lower, but rates of inversion fixation remain higher on the Y chromosome than on autosomes across a large range of parameter values. Furthermore, they also stated that s =  0 is not a standard or appropriate control for studies on inversion dynamics when deleterious mutations are segregating.

PLOS consulted two experts in the field on these issues, who evaluated the article [1], the scientific critiques raised by Olito and Charlesworth [3], and the authors’ responses.

The first expert concluded that concerns raised in [3] are valid. Specifically, they agreed with the analysis presented in the critique [3], and advised that the s =  0 case is relevant to support the inference that differences observed between relative rates of inversion fixation on autosomes and Y chromosomes may be attributed to the sheltering process, with s =  0 establishing bounds under which the sheltering effect cannot be employed as an explanation. It was noted that the article [1] did not report a figure capturing the realistic bounds of the proposed sheltering process and that the results are likely to be interpreted incorrectly by readers. Although the consulted expert did not identify any errors in the mathematical results reported in the article [1], they advised that important claims and inferences in the article are not supported. They further advised that the authors’ responses did not satisfactorily resolve these issues which are critical to the article’s main claims about the breadth of parameter space under which the sheltering process can operate.

The second expert concurred with the concerns about the claimed breadth of parameter space, and advised that the published figures in the article [1] are not sufficient to quantify the strength and generality of the sheltering process and that studying the transition towards and including the neutral case (s =  0) is useful.

The *PLOS Biology* Editors concluded that some findings reported in the article may be valid, but key aspects of the conclusions are not adequately supported by the published analyses and so the article does not meet *PLOS Biology*’s publication criteria. Therefore, the *PLOS Biology* Editors retract this article.

All authors stand by the article’s findings and did not agree with the editorial assessment or the retraction decision.

*Note: Additional discourse on this matter is available elsewhere [*3–5*]. A detailed critique posted as a pre-print [*3*] was used in PLOS Biology’s editorial assessment; further discussion of the sheltering hypothesis was later published in a perspective article [*4*]; and the authors of [*1*] recently posted a pre-print [*5*] in response to issues raised in [*3*]. PLOS does not endorse any allegations or discourse in [*3*–*5*].*
